# A PARP1-related prognostic signature constructing and PARP-1 inhibitors screening for glioma

**DOI:** 10.3389/fcell.2022.916415

**Published:** 2022-08-24

**Authors:** Hui Li, Zhenhua Wang, Yuanyuan Hou, Jianxin Xi, Zhenqiang He, Han Lu, Zhishan Du, Sheng Zhong, Qunying Yang

**Affiliations:** ^1^ Department of Neurosurgery/Neuro-oncology, Sun Yat-sen University Cancer Center, State Key Laboratory of Oncology in South China, Collaborative Innovation Center for Cancer Medicine, Guangzhou, China; ^2^ Department of Neurology, The First Hospital of Jilin University, Changchun, China; ^3^ Clinical College, Jilin University, Changchun, China

**Keywords:** Glioma, PARP-1 (Poly ADP-ribose polymerase 1), DNA damage repair, Inhibitors, drug screening

## Abstract

The current standard treatments of glioma include surgical resection, supplemented with radiotherapy and chemotherapy, but the prognosis is poor. PARP-1 (Poly ADP-ribose polymerase 1) is a hot spot for cancer-targeted therapy and was reported to be significantly elevated in glioma. In this study, we analyzed the role of PARP-1 in DNA damage repair, constructed a PARP1-related DNA-repair prognostic signature (DPS), and screened targeted drugs for glioma. RNA-seq data of 639 glioma samples were downloaded from the GEO (Gene Expression Omnibus) database and divided into PARP1_H and PARP1_L according to the front and rear thirds of the expression level of PARP-1. First, we systematically analyzed the influence of PARP-1 on DNA damage repair, prognosis, and chemoradiotherapy sensitization of glioma. All glioma patients and patients with radiotherapy or chemotherapy had a better prognosis in PARP1_L than in PARP1_H. Next, differentially expressed DNA-repair related genes (DEGs) were identified between PARP1_H and PARP1_L by LASSO (Least Absolute Shrinkage and Selection Operator) Cox analysis and applied for constructing DPS. Based on the four-gene DPS, we then developed a new nomogram to assess overall survival in glioma patients. Additionally, PARP-1 was proved an effective target for glioma therapy. So, a series of computer-aided techniques, including Discovery Studio 4.5, Schrodinger, and PyMol, were applied for the virtual screening of favorable PARP-1 inhibitors. In conclusion, this study investigated the effect of PARP-1 on glioma prognosis and the sensitization effect of radiotherapy and chemotherapy, established a novel nomogram to evaluate the overall survival of glioma patients, and further explored targeted therapy for glioma.

## Introduction

Glioma is the most common primary intracranial malignancy. And current standard treatments include surgical resection, supplemented by radiotherapy and chemotherapy, but the prognosis is poor (Ames and Gold, 1991; Prokhorova et al., 2021). A lot of experience has been accumulated in tumor electric field therapy, immunotherapy, and targeted molecular therapy for glioma, but few results can truly change clinical practice (Stupp et al., 2017; Fangusaro et al., 2019; Hargrave et al., 2019; Reardon et al., 2020). Therefore, it is necessary to more accurately predict the prognosis of glioma patients and develop more effective treatments.

**FIGURE 1:**
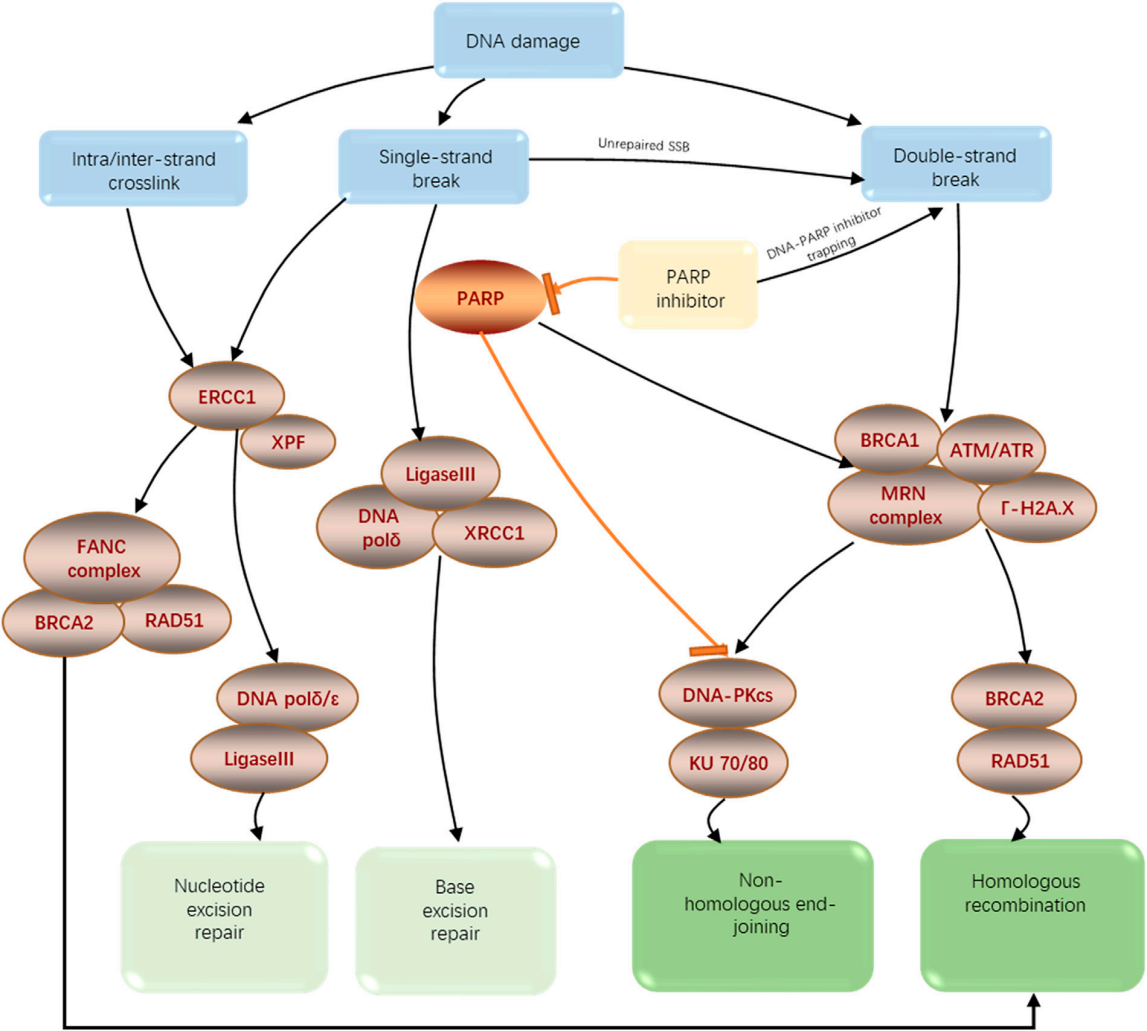
Schematic diagram of the DNA damage repair pathway.

Genomic instability is one of the most prevalent features of tumor cells. It may be the combined effect of DNA damage, tumor-specific DNA repair defects, and failure to halt the cell cycle before the delivery of damaged DNA. Although these processes lead to genomic instability and disease processes, they also offer therapeutic opportunities (Lord and Ashworth, 2012) (Hannigan et al., 2013). DNA damage repair (DDR) determines not only the occurrence and development of tumors but also the sensitivity or tolerance of tumor cells to radiotherapy, chemotherapy, and other treatments that induce DNA damage. So, dysregulation of DDR pathways plays a significant part in tumor prognosis prediction and treatment. There are many ways of DDR, including nucleotide excision repair (NER), non-homologous end joining (NHEJ), homologous recombination (HR), mismatch repair (MMR), base excision repair (BER) (Figure 1), etc. DNA double-strand breaks are the most cytotoxic damage, and homologous recombination repair (HRR) is the most important and accurate repair method in DNA double-strand damage repair (Lord and Ashworth, 2012).

PARP (poly ADP-ribose polymerase) family are enzymes that catalyze the PAR modification of proteins and other substances (Kumar et al., 2020). Among them, PARP-1 was the most widely studied and played a key role in DNA repair pathways, especially in HRR (10, 11). When cellular DNA is damaged, PARP-1 responds immediately. It rapidly binds to the damaged DNA and catalyzes the decomposition of NAD + into nicotinamide and ADP (adenosine diphosphate). Then, ADP is linked to the self-modified region of PARP-1 and undergoes a complex reaction of poly-ADP-ribose to form PAR (Poly ADP-ribose). When reaching a certain length, the PAR poly chain dissociates from the DNA and then guides DNA repair enzymes such as XRCC 1 (X-ray repair cross-complementary gene 1) and DNA ligase III to carry out BER to remove the wrong or damaged bases (Gorren et al., 1997; Sandhu et al., 2013). Next, new single-stranded DNA fragments are synthesized by DNA polymerase and ligated with the original single-stranded DNA by ligase to complete DNA damage repair (Ossovskaya et al., 2010).

Currently, the research reports on PARP-1 mainly focus on its expression and function in tumors lacking the BRCA1/2 gene, such as ovarian cancer, metastatic breast cancer, advanced prostate cancer, and pancreatic cancer (Ledermann et al., 2015; Robson et al., 2017; Golan et al., 2019; Abida et al., 2020). Recently, a study found that the expression level of PARP-1 mRNA in glioma cell lines was significantly increased, suggesting that PARP-1 is expected to become a new prognostic indicator for glioma patients and a new anti-glioma therapy target (Han et al., 2020).

In this study, RNA-seq data of 639 glioma samples were downloaded from the GEO (Gene Expression Omnibus) database and divided into PARP1_H and PARP1_L according to the front and rear thirds of the expression level of PARP-1. First, we systematically analyzed the influence of PARP-1 on DNA damage repair, prognosis, and chemoradiotherapy sensitization of glioma. Next, differentially expressed DNA-repair related genes (DEGs) were identified between PARP1_H and PARP1_L by LASSO (Least Absolute Shrinkage and Selection Operator) Cox analysis and applied to the construction of DPS. Based on the four-gene DPS, we then developed a new nomogram to assess overall survival in glioma patients. Additionally, a series of computer-aided techniques were applied for screening potential inhibitors for the DNA damage repair pathway. In conclusion, this study investigated the effect of PARP-1 on glioma prognosis and the sensitization effect of radiotherapy and chemotherapy, established a novel nomogram to evaluate the overall survival of glioma patients, and further explored targeted therapy for glioma.

## Methods and materials

### Gene expression datasets, data processing, and functional enrichment analysis

We acquired RNA-seq data of 639 glioma samples from the GEO (Gene Expression Omnibus) database. The survival data were obtained for all patients. According to the front and rear thirds of the expression level of PARP-1, the patients were divided into PARP1_H (*n* = 213) and PARP1_L (*n* = 213). Additionally, the RNA transcriptome analysis was carried out by transformation of log2-based FPKM values.

A total of 329 genes of DNA-repair proteins were downloaded from the PathCards website (https://pathcards.genecards.org/). To analyze signalling pathway enrichment, 278 genes of these genes detected in glioma patients were uploaded to Metascape (https://metascape.org/) to identify GO (Gene Ontology) Terms and KEGG (Kyoto Encyclopedia of Genes and Genomes) pathways (Zhou et al., 2019). Terms were thought significant when the conditions of *p* < 0.01 and the number of enriched genes ≥3 were met and grouped separately according to their membership similarity. And the term with the best *p*-value in every 20 clusters was selected.

### Prognosis and gene enrichment between PARP1_H and PARP1_L

We systematically analyzed the influence of PARP-1 on DNA damage repair, prognosis, and chemoradiotherapy sensitization of glioma. The OS (overall survival) of glioma patients was compared between PARP1_H and PARP1_L, PARP1_H and PARP1_L with chemotherapy, PARP1_H and PARP1_L with radiotherapy, and PARP1_H and PARP1_L with both chemotherapy and radiotherapy. Kaplan-Meier curves were drawn to show differences in survival time. The log-rank test was carried out to evaluate the significance of differences in survival times with a threshold of *p* < 0.05. In addition, expression levels of the 278 genes of the DNA-repair proteins were compared between PARP1_H and PARP1_L.

### Differential expression analysis

Differential expression analysis was calculated and carried out on RStudio by the Wilcoxon Rank Sum and Signed Rank Tests between PARP1_H and PARP1_L (Servant et al., 2010). The limma package of RStudio software was applied to distinguish the differentially expressed DNA-repair related genes (DEGs) between PARP1_H and PARP1_L. Genes with log2 |fold change| ≥1 and FDR (False Discovery Rate) < 0.05 were chosen as DEGs (differentially expressed genes). Then, the ImmPort database identifies differentially expressed genes for DNA-repair proteins (DEGs) (https://www.immport.org/). Patients were divided into High (*n* = 213) and Low (*n* = 213) groups based on the median expression level of each DEG.

The OS of glioma patients was compared between the High and Low groups of each DEDG. Kaplan-Meier curves were drawn to show differences in survival time. The log-rank test was carried out to evaluate the significance of differences in survival times with a threshold of *p* < 0.05.

Construction of the PARP1-related DNA-repair prognostic signature (DPS).

To build the PARP1-related DNA-repair prognostic signature (DPS), DEGs were put in LASSO Cox regression and analyzed by the “glmnet” R package (Tibshirani, 1997) (Friedman et al., 2010). The DPS model was constructed from weighted Cox regression coefficients to estimate the risk score for each patient. Patients were classified as high or low risk according to the best cutoff values obtained by the “survminer” R package. We used the “survival ROC” R package to generate ROC (Receiver Operating Characteristic) curves (Heagerty et al., 2000). And the area under the curve values of the ROC curve was calculated to assess the specificity and sensitivity of DPS.

### Development of the nomogram

Univariate and multivariate Cox analyses were applied to assess the independent prognostic ability of DPS. Then we performed the “rms” package to construct an innovative nomogram according to the Cox analysis results. To determine the accuracy, calibration plots of observed vs. predicted probabilities of 1-, 3-, and 5-years OS were developed. The C-Index (Concordance Index) was calculated to determine the discriminative power of the model. And the C- index was corrected using bootstraps.

### Virtual screening of PARP-1 inhibitors using libdock, ADME and TOPKAT

DS 4.5 (Discovery Studio 4.5, Accelrys, Inc.) is a suite of software for modelling large and small-molecule systems. Libdock, ADME (absorption, distribution, metabolism, excretion), and TOPKAT (Toxicity Prediction by Computer Assisted Technology) modules of DS 4.5 were used for virtual screening firstly. Libdock is a rigid-based docking program. During this procedure, hotspots for PARP-1 were calculated, and the ligands formed favorable interactions based on the hotspots. Afterward, poses of all the ligands were ranked according to their Libdock scores. The 3.22 Å crystal structure of PARP-1 in complex with inhibitor (PJ34L) was downloaded from PDB (Protein Database) (https://www.rcsb.org). NAD + binds to PARP-1 and is catalyzed to ADP ribose in this region. So, the binding pocket was chosen as the docking region for screening. Moreover, 17,799 natural, named, and purchasable molecules were downloaded from the ZINC15 database for virtual screening (https://zinc.docking.org/). And Lynparza (also called Olaparib) was selected as a reference inhibitor (Brooks et al., 2009). Only the top 20 molecules were chosen for the ADME and TOPKAT analysis. The ADME module was applied for calculating the pharmacological properties of selected compounds and Lynparza, including the absorption, distribution, metabolism, and excretion. TOPKAT module was used to assess the toxicological properties. Finally, two molecules were chosen as favorable candidates based on the above results.

### Precise molecular docking using CDOCKER

Precise docking was performed between selected compounds, Lynparza, and prepared PARP-1 by CDOCKER module of DS 4.5 based on CHARMm36 force field. The receptor is held rigid while the ligands flex during the docking process. The CDOCKER interaction energy indicating ligand binding affinity was calculated for each complex pose. The binding site sphere of PARP-1 was defined as the region within a radius of 13 Å from the geometric centroid of Lynparza. Ligands can bind to residues within the binding site sphere during the docking process. Different poses of each test molecule were generated, and their CDOCKER interaction energies were analyzed separately. Schrodinger and PyMol software were used to visualize further the optimal postural binding of selected compounds, Lynparza and PARP-1.

### Pharmacophore analysis and molecular dynamics simulation

Pharmacophores of selected compounds and Lynparza were analyzed by the 3D-QSAR module of DS 4.5. Only those with energies below 10 kcal/mol can be retained, and a maximum of 255 confirmations can be generated per molecule.

In addition, to assess the stability and affinity of each compound-PARP-1 complex in the natural environment, the best binding conformation was selected and prepared for Molecular dynamics simulation. The ligand- PARP-1 complex was placed in an orthorhombic box and solvated using an explicit periodic boundary solvation water model. To simulate the physiological environment of the system, solidum chloride was added. The production procedure was carried out for 100 ps and the time step was 2 fs. Concerning the initial complex setup, the trajectory protocol of DS 4.5 was performed to determine the trajectory for potential energy and RMSD (root-mean-square deviation).

## Result

### Functional enrichment analysis

**FIGURE 2:**
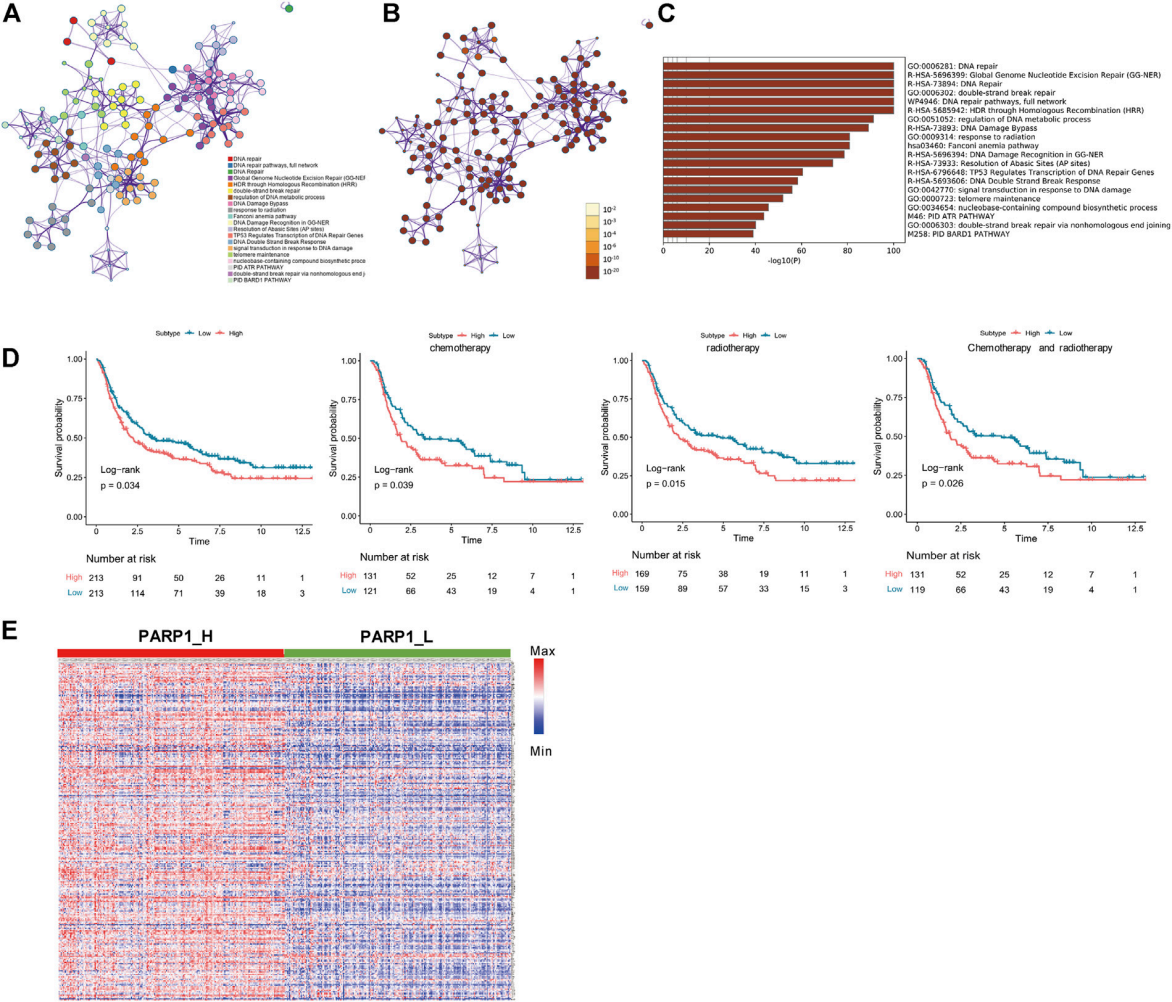
Functional enrichment analysis of 278 genes of the DNA-repair proteins, and analyses between PARP1_L (*n* = 213) and PARP1_L (*n* = 213) from GEO database. **(A)** Enriched terms are colored by cluster ID, where nodes that share the same cluster ID are typically close to each other in 278 genes of the DNA-repair proteins. **(B)** Enriched terms are colored by *p*-value, where terms containing more genes have a more significant *p*-value in 278 genes of the DNA-repair proteins. **(C)** Heatmap of enriched terms across input gene lists, colored by *p*-values. **(D)** Comparison of survival prognosis between PARP1_H and PARP1_L, between PARP1_H and PARP1_L after chemotherapy, between PARP1_H and PARP1_L after radiotherapy, and between PARP1_H and PARP1_L after chemotherapy and radiotherapy from GEO using the Log-Rank test. **(E)** expression levels of the 278 genes of the DNA-repair proteins were compared between PARP1_H and PARP1_L.

The 278 genes of the DNA-repair proteins were mainly enriched in GO:0006281: DNA repair, GO:0006302: double-strand break repair (GO terms), R-HSA-73894: DNA Repair, R-HSA-5685942: HDR through Homologous Recombination (HRR), R-HSA-5696399: Global Genome Nucleotide Excision Repair (GG-NER), WP4946: “DNA repair pathways, full network” (KEGG). Each node represents a collective term, colored first by cluster ID and its *p*-value, separately (Figures 2A–C).

### Comparison of prognosis and gene expression between PARP1_H and PARP1_L

Survival analysis indicated that the clinical prognoses of PARP1_H and PARP1_L were different. All glioma patients, patients with radiotherapy, patients with chemotherapy, and patients with both radiotherapy and chemotherapy had a better prognosis in PARP1_L than PARP1_H (Log-Rank test, P (all) = 0.034; P (radiotherapy) = 0.015; P (chemotherapy) = 0.039; P (radiotherapy and chemotherapy) = 0.026) (Figure 2D). Additionally, as shown in Figure 2E, the expression levels of 278 DNA repair-related protein genes were significantly higher in PARP1_H than in PARP1_L.

Discrimination against differentially expressed DNA-repair related genes (DEGs).

**FIGURE 3:**
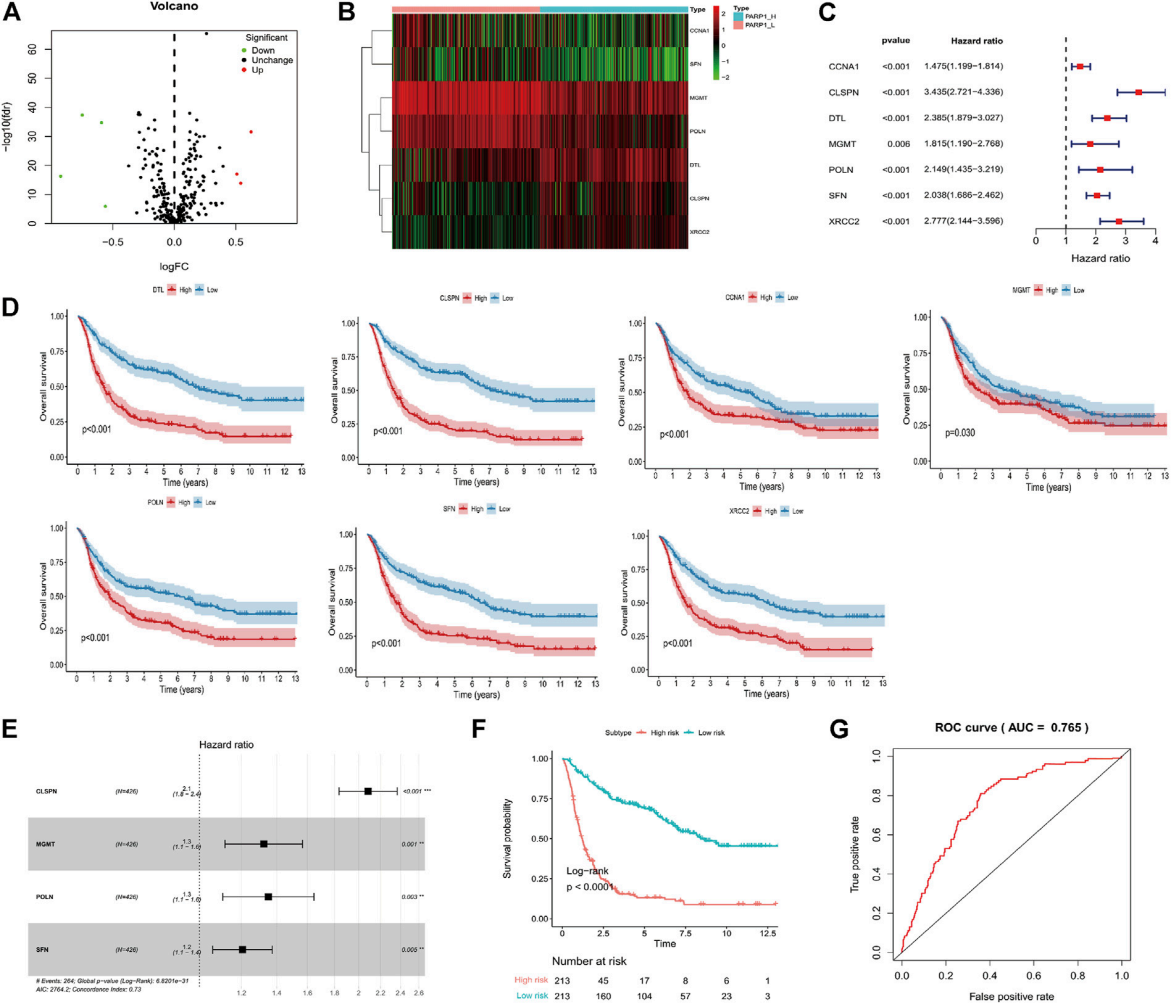
Identification of PARP1-related differentially expressed genes of DNA-repair proteins, and construction of the PARP1-related DNA-repair prognostic signature (DPS). **(A)** Volcano plot of 7 DNA-repair proteins differentially expressed between PARP1_L (*n* = 213) and PARP1_L (*n* = 213). **(B)** Heatmap of genes of DNA-repair proteins differentially expressed between PARP1_H (*n* = 213) and PARP1_L (*n* = 213) **(C,D)**. The relationship between DEG expression level and prognosis of glioma patients. The OS of glioma patients was compared between High (*n* = 213) and Low (*n* = 213) groups of each DEG using the Log-Rank test. **(E)** LASSO Cox analysis identified four genes most correlated with overall survival. **(F)** Kaplan-Meier curves of overall survival based on the DPS [n (high risk) = 213, n (low risk) = 213]. **(G)** ROC curve analysis of the DPS.

Seven genes were confirmed according to the standard (log2 |fold change| ≥1 and FDR <0.05), of which 3 genes were up-regulated, and four were down-regulated (Figure 3A). Seven DEGs were chosen using the ImmPort database for performing prognostic analysis Gene expression patterns are shown in Figure 3B. For each DEG, patients were divided into High (*n* = 213) and Low (*n* = 213) according to their median expression level. Then, the OS of glioma patients was compared between the High and Low groups of each DEG Figure 3C. Patients of each DEG’s High group had a worse prognosis than that of the Low group (Log-Rank test, CCNA1: *p* < 0.001, CLSPN: *p* < 0.001, DTL: *p* < 0.001, MGMT: *p* = 0.03, POLN: *p* < 0.001, SFN: *p* < 0.001, XRCC2: *p* < 0.001) (Figure 3D).

### Construction of the PARP-1-related DNA-repair prognostic signature

LASSO Cox regression analysis of DEGs was used to construct a PARP1-related DNA-repair prognostic signature (DPS) (Figure 3E). Risk scores were evaluated for each glioma patient (risk score = CLSPN*0.734 + MGMT*0.28 + POLN*0.3 + SFN*0.187). Patients were divided into low-risk and high-risk groups according to the optimal cutoff value (0.89005466) evaluated by the “survminer” R package. Kaplan-Meier analysis showed that patients with low-risk scores had better outcomes than those with high-risk scores (Figure 3F). The ROC curve analysis of the DPS suggested good prognostic ability for OS (Figure 3G).

### Establishment of a DPS-based nomogram model

**FIGURE 4:**
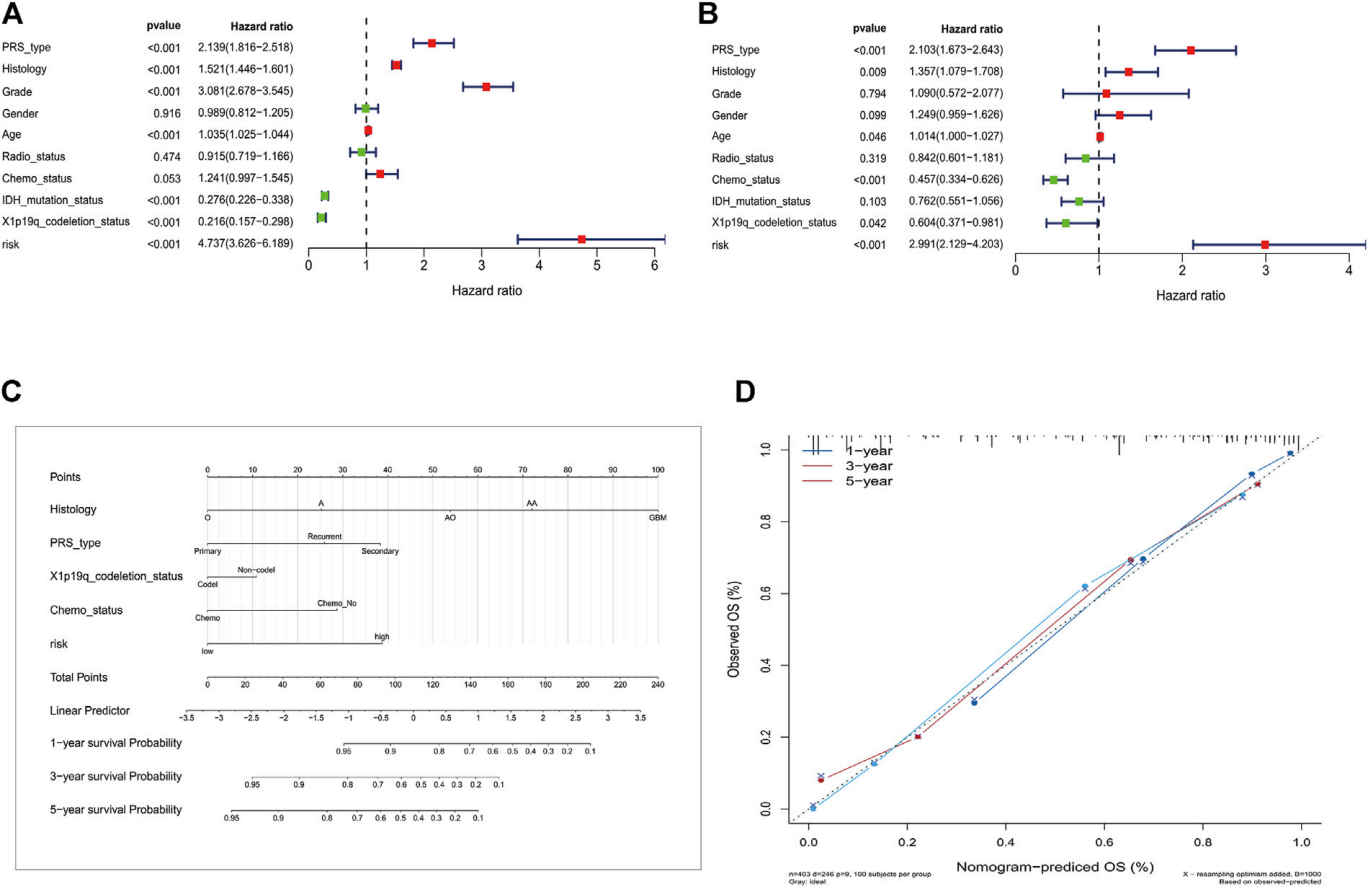
Construction of the nomogram model. **(A)** Univariate and multivariate Cox analyses indicating that the DPS is significantly associated with OS. **(B)** Multivariate Cox analyses indicating that the DPS is significantly associated with OS. **(C)** Nomogram model for predicting the probability of 1-, 3-, and 5- year OS in Gliomas patients. **(D)** Calibration plots of the nomogram for predicting the probability of OS at 1, 3, and 5 years.

The DPS was indicated to be significantly associated with OS (Hazard ratio: 4.737%, 95% confidence interval: 3.626–6.189, *p* < 0.001) by the univariate Cox analysis (Figure 4A). From the multivariate Cox analysis, DPS proved to be an independent prognostic factor (Hazard ratio: 2.739, 95% confidence interval: 1.923–3.902, *p* < 0.001) (Figure 4B). Finally, a DPS-based nomogram model was established Figure 4C. The C-index was 0.674, which revealed the specific discriminative ability of the nomogram model. Moreover, the observed vs predicted probabilities of 1-, 3-, and 5-years OS showed good agreement in the calibration plot (Figure 4D).

### Virtual screening using libdock, ADME, and TOPKAT of DS 4.5

**FIGURE 5:**
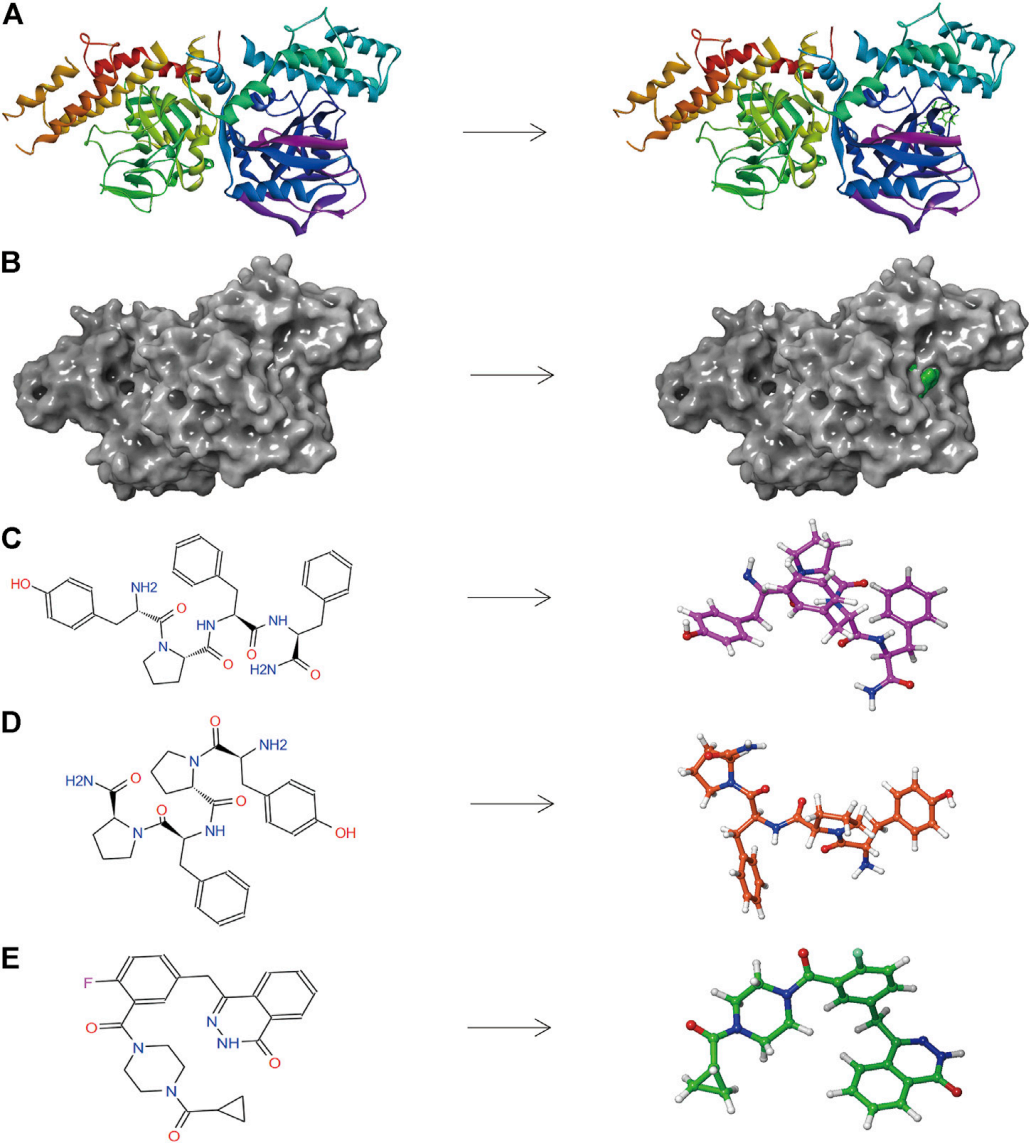
**(A)** The molecular structure of PARP-1 and the complex structure of PARP-1 with Lynparza. Initial molecular structure was shown. **(B)** The molecular structure of PARP-1 and the complex structure of PARP-1 with Lynparza. The surface of the complex was added, green for Lynparza and gray for PARP-1. **(C)** 2D and 3D chemical structures of ZINC000014951634. **(D)** 2D and 3D chemical structures of ZINC000053057130. **(E)** 2D and 3D chemical structures of Lynparza.

Based on the above results, PARP-1 was proved an essential target for glioma therapy and prognosis. Therefore, we took PARP-1 as the target for further drug screening. The 3D (three-dimensional) structures of PARP-1 and the Lynparza-PARP1 complex are displayed in Figures 5A, B. According to Libdock’s results, 2,996 compounds were identified that stably bind to PARP-1. Among them, 37 molecules had higher Libdock scores than Lynparza (ranking: 38, Libdock score: 153.31). Table 1 lists the top 20 compounds by Libdock scores.

**TABLE 1:** Top 20 ranked compounds with higher libdock scores than lynparza.

Number	Compounds	Libdock score	Number	Compounds	Libdock score
1	ZINC000003995616	197.589	11	ZINC000021992902	169.035
2	ZINC000011616634	183.062	12	ZINC000012495612	165.482
3	ZINC000011616633	180.699	13	ZINC000031298217	162.833
4	ZINC000017654900	179.771	14	ZINC000044306670	162.746
5	ZINC000028968107	173.664	15	ZINC000003979028	162.196
6	ZINC000049872065	172.943	16	ZINC000002033589	161.044
7	ZINC000002528509	171.533	17	ZINC000044086691	160.416
8	ZINC000073280937	171.524	18	ZINC000034944433	159.795
9	ZINC000014951634	170.928	19	ZINC000038143594	159.372
10	ZINC000053057130	170.314	20	ZINC000002528486	158.692

The pharmacological and toxicological properties of the top 20 compounds and Lynparza were evaluated by the ADME (Table 2) and TOPKAT (Table 3) modules of DS 4.5. Compounds 1 (ZINC000014951634) and 2 (ZINC000053057130) showed no hepatotoxicity, non-CYP2D6 inhibitor, low Ames mutagenicity, low rodent carcinogenicity, and low developmental toxicity potential, which strongly suggests their promising application in drug development. So, compounds 1 and 2 were chosen as favorable inhibitors of PARP-1. The 3D and two-dimensional (2D) chemical structures of compounds 1, 2 and Lynparza are shown in Figures 5C–E.

**TABLE 2:** ADME (Adsorption, Distribution, Metabolism, Excretion) properties of compounds.

Number	Compounds	Solubility Level^a^	BBB level^b^	CYP2D6^c^	Hepatotoxicity^d^	Absorption Level^e^	PPB Level^f^
1	ZINC000003995616	1	4	0	0	2	1
2	ZINC000011616634	2	4	0	0	3	0
3	ZINC000011616633	2	4	0	0	3	0
4	ZINC000017654900	2	4	0	1	2	0
5	ZINC000028968107	1	4	1	1	3	1
6	ZINC000049872065	3	4	0	0	2	0
7	ZINC000002528509	2	4	1	1	0	1
8	ZINC000073280937	2	4	0	1	2	1
9	ZINC000014951634	3	4	0	0	3	0
10	ZINC000053057130	3	4	0	0	3	0
11	ZINC000021992902	3	4	0	0	1	0
12	ZINC000012495612	3	4	0	1	3	0
13	ZINC000031298217	2	4	0	1	2	0
14	ZINC000044306670	2	4	0	0	3	1
15	ZINC000003979028	2	4	0	1	3	0
16	ZINC000002033589	2	4	1	0	3	0
17	ZINC000044086691	1	4	0	0	3	1
18	ZINC000034944433	2	4	1	0	2	0
19	ZINC000038143594	3	4	0	0	3	0
20	ZINC000002528486	2	2	1	1	0	1
21	Lynparza	3	3	0	1	0	1

a Aqueous-solubility level: 0 (extremely low); 1 (very low, but possible); 2 (low); 3 (good).

b Blood Brain Barrier level: 0 (Very high penetrant); 1 (High); 2 (Medium); 3 (Low); 4 (Undefined).

c Cytochrome P450 2D6 level: 0 (Non-inhibitor); 1 (Inhibitor).

d Hepatotoxicity: 0 (Nontoxic); 1 (Toxic).

e Human-intestinal absorption level: 0 (good); 1 (moderate); 2 (poor); 3 (very poor).

f Plasma Protein Binding: 0 (Absorbent weak); 1 (Absorbent strong).

**TABLE 3:** Toxicities of compounds.

Number	Compounds	Mouse NTP^a^		Rat NTP^a^		AMES^b^	DTP^c^
		Female	Male	Female	Male		
1	ZINC000003995616	0.235	0.002	0.245	0.300	0.004	0.252
2	ZINC000011616634	0.761	0.509	0.308	0.583	0.000	0.493
3	ZINC000011616633	0.761	0.509	0.308	0.583	0.000	0.493
4	ZINC000017654900	0.572	0.005	0.162	0.517	0.000	0.321
5	ZINC000028968107	0.110	0.321	0.336	0.045	0.115	0.629
6	ZINC000049872065	0.576	0.611	0.215	0.525	0.000	0.793
7	ZINC000002528509	0.299	0.439	0.422	0.483	0.000	0.618
8	ZINC000073280937	0.802	0.873	0.476	0.290	0.012	0.502
9	ZINC000014951634	0.136	0.016	0.228	0.482	0.001	0.462
10	ZINC000053057130	0.157	0.005	0.223	0.465	0.000	0.437
11	ZINC000021992902	0.578	0.614	0.220	0.492	0.001	0.764
12	ZINC000012495612	0.475	0.574	0.309	0.653	0.075	0.834
13	ZINC000031298217	0.218	0.552	0.523	0.566	0.593	0.677
14	ZINC000044306670	0.385	0.614	0.411	0.146	0.126	0.780
15	ZINC000003979028	0.479	0.482	0.494	0.748	0.511	0.660
16	ZINC000002033589	0.470	0.348	0.325	0.486	0.002	0.856
17	ZINC000044086691	0.562	0.829	0.193	0.281	0.031	0.823
18	ZINC000034944433	0.502	0.433	0.327	0.526	0.002	0.836
19	ZINC000038143594	0.384	0.405	0.265	0.300	0.178	0.614
20	ZINC000002528486	0.275	0.564	0.462	0.443	0.000	0.587
21	Lynparza	0.665	0.311	0.440	0.627	0.368	0.672

a <0.3 (Non-Carcinogen); >0.7 (Carcinogen).

b < 0.3 (Non-Mutagen); >0.7 (Mutagen).

c < 0.3 (Non-Toxic); >0.7 (Toxic).

### Ligand binding analysis

**FIGURE 6:**
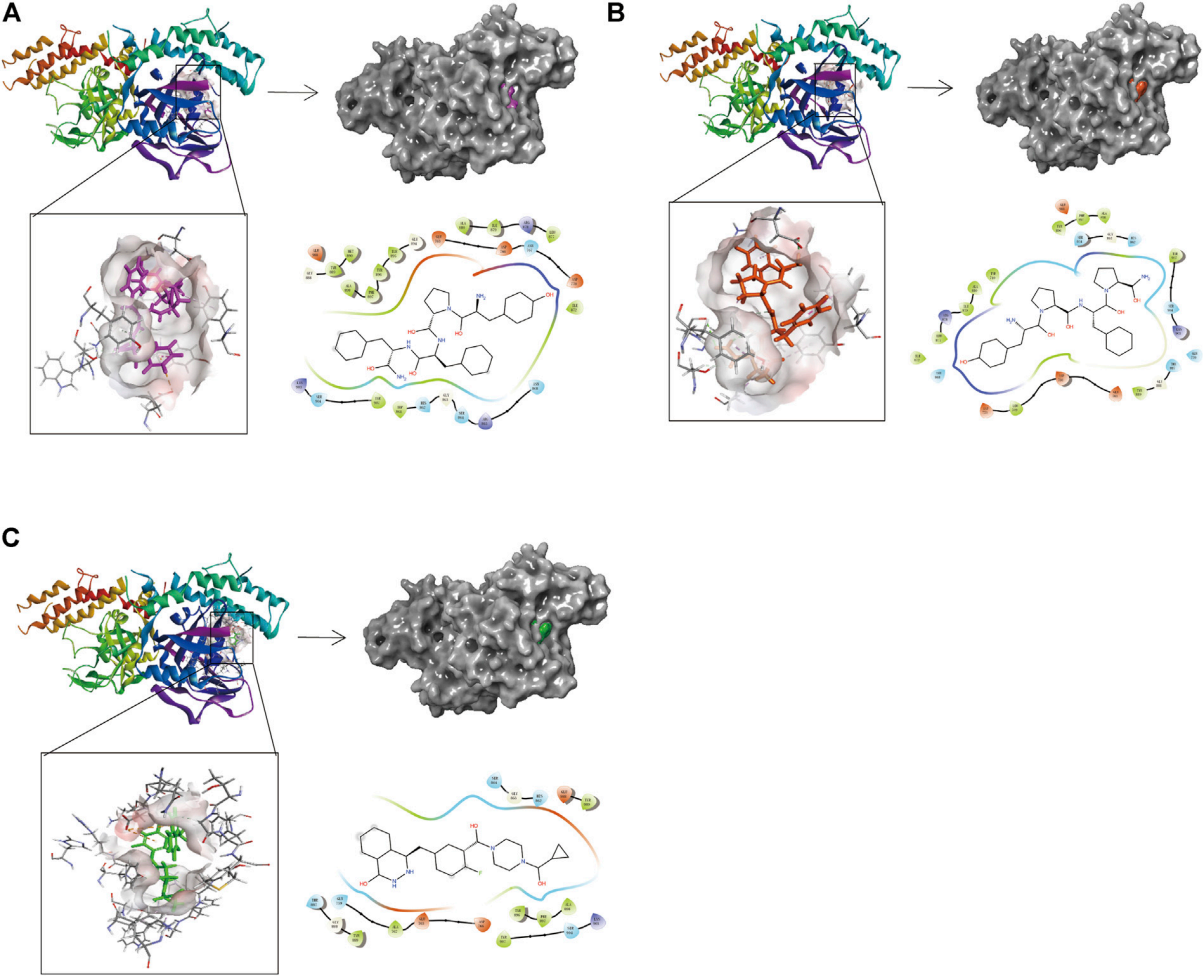
The 3D and 2D schematic drawing of interactions between ligands and PARP-1 by DS 4.5, and Schrodinger. The surface of the binding area was added; blue represented positive charge; red represented negative charge; ligands were shown in sticks; the structure around the ligand-receptor junction was shown in thinner sticks. In addition, the surface of the complex was added, purple for ZINC000014951634, orange for ZINC000053057130, green for Lynparza and gray for PARP-1. **(A)** ZINC000014951634- PARP-1 complex; **(B)** ZINC000053057130- PARP-1 complex; **(C)** Lynparza -PARP-1 complex.

**FIGURE 7:**
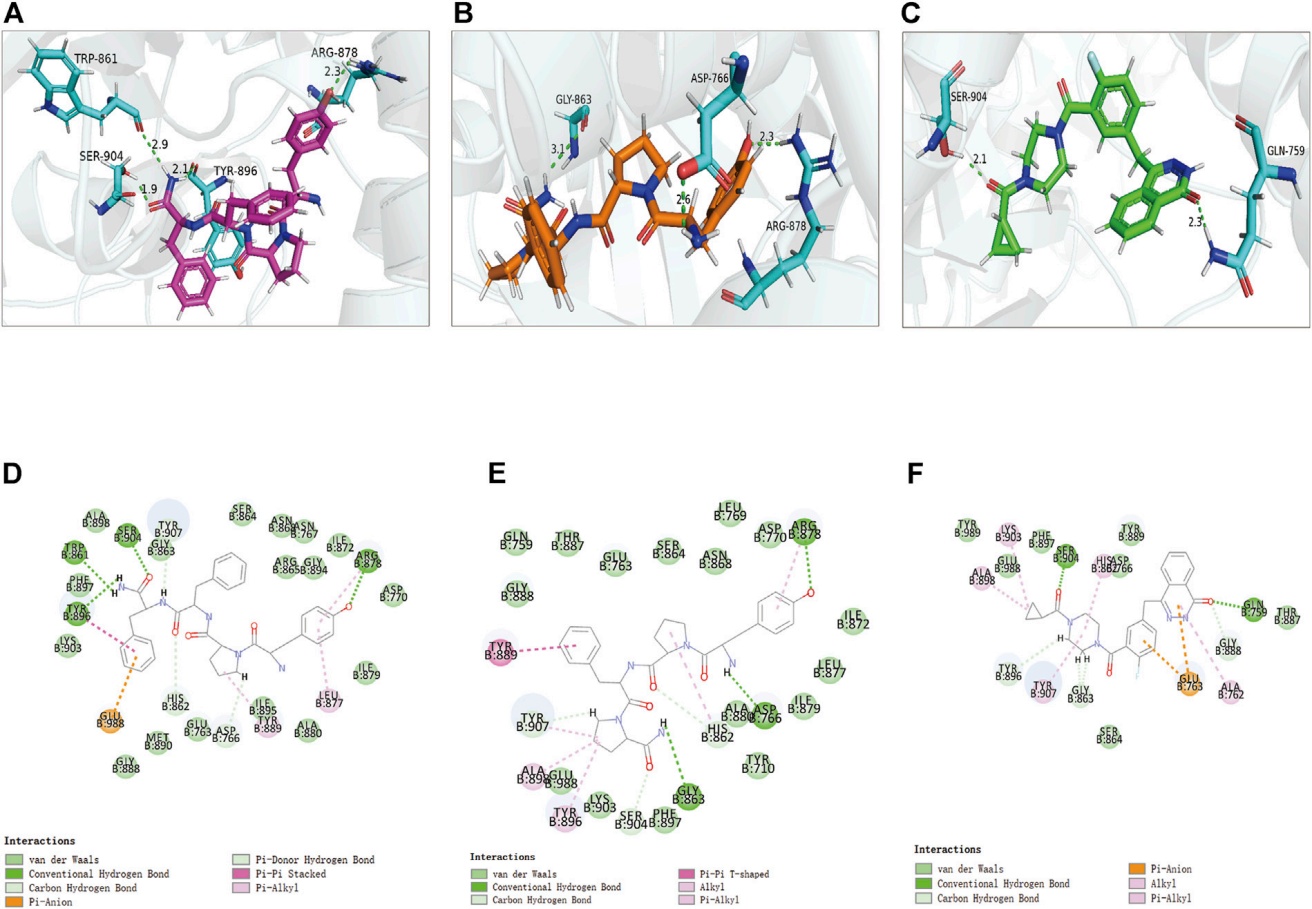
The 3D and 2D intermolecular interactions in the binding pockets by Schrodinger and PyMol of ligand-PARP-1 complex. Green represents Hydrogen bond. **(A–D)** ZINC000014951634- PARP-1 complex; **(B–E)** ZINC000053057130- PARP-1 complex; **(C–F)** Lynparza -PARP-1 complex.

Compounds 1, 2, and Lynparza were precisely docked into the function pocket of PARP-1 by the CDOCKER module (Figures 6, 7). Table 4 showed that the CDOCKER interaction energies of compounds 1, 2 were significantly lower than that of the reference ligand Lynparza (-54.2416 kcal/mol), indicating that these two compounds have higher stability and affinity with PARP-1 than Lynparza.

**TABLE 4:** | CDOCKER interaction energy of compounds with PARP-1.

Compounds	CDOCKER interaction energy (Kcal/mol)
ZINC000014951634	−72.8455
ZINC000053057130	−70.3196
Lynparza	−54.2416

Structural analyses of the ligands-PARP-1 complex were also performed for the hydrogen bonds, Pi-Pi interaction, Pi-Alkyl interaction, Pi-Anion interaction, and Alkyl interactions (Figures 6, 7 and Table 5, 6). Results showed that compound 1 formed four pairs of hydrogen bonds, one pair of Pi-Pi staked interaction, three pairs of Pi-Alkyl interaction, and one pair of Pi-Anion interaction with PARP-1. Compound 2 formed three pairs of hydrogen bonds, one pair of Pi-Pi T-shaped interactions, one pair of Pi-Alkyl interactions, and one pair of Alkyl interactions with PARP-1. The reference compound Lynparza formed two hydrogen bonds, three pairs of Pi-Alkyl interactions, and two pairs of Pi-Anion interactions with PARP-1. In addition, Schrodinger and PyMol software were used to analyze further and visualize the interaction between the ligand and PARP-1 in the binding pocket.

**TABLE 5:** Hydrogen bond interaction parameters for each compound and PARP-1 residues.

Receptor	Compound	Donor atom	Receptor atom	Distances (Å)
PARP-1	ZINC000014951634	B:ARG878:HH21	ZINC000014951634:O40	2.3
		B:TYR896:O	ZINC000014951634:H44	2.1
		B:TRP861:O	ZINC000014951634:H43	2.9
		B:SER904:HG	ZINC000014951634:O3	1.9
	ZINC000053057130	B:GLY863:O	ZINC000053057130:H39	3.1
		B:ASP766:OD1	ZINC000053057130:H66	2.1
		B:ARG878:HH21	ZINC000053057130:O36	2.3
	Lynparza	B:SER904:HG	Molecule:O27	2.1
		B:GLN759:HE21	Molecule:O11	2.3

**TABLE 6:** Pi-Pi interaction, Pi-Alkyl interaction, Pi-Anion interaction and Alkyl interaction parameters for each compound and PARP-1 residues.

Interaction parameters	Receptor	Compound	Donor atom	Receptor Atom	Distances (Å)
Pi-Pi staked interaction	PARP-1	ZINC000014951634	B:TYR896	ZINC000014951634	4.62
Pi-Pi T-shaped interaction		ZINC000053057130	B:TYR889	ZINC000053057130	5.08
Pi-Alkyl interaction		ZINC000014951634	B:TYR889	ZINC000014951634	4.95
			B:ARG878	ZINC000014951634	5.32
			B:LEU877	ZINC000014951634	5.04
		ZINC000053057130	B:TYR896	ZINC000053057130	5.49
			B:TYR907	ZINC000053057130	4.97
			B:HIS862	ZINC000053057130	5.26
			B:ARG878	ZINC000053057130	4.93
		Lynparza	B:TYR907	Molecule	4.00
			B:HIS862	Molecule	4.77
			B:ALA762	Molecule	5.47
Pi-Anion interaction		ZINC000014951634	B:GLU988:OE1	ZINC000014951634	4.18
		Lynparza	B:GLU763:OE2	Molecule	4.52
			B:GLU763:OE2	Molecule	4.87
Alkyl interaction		ZINC000053057130	B:ALA898	ZINC000053057130	5.17
		Lynparza	B:ALA898	Molecule	4.49
			B:LYS903	Molecule	4.86

### Pharmacophore analysis and molecular dynamics simulation

**FIGURE 8:**
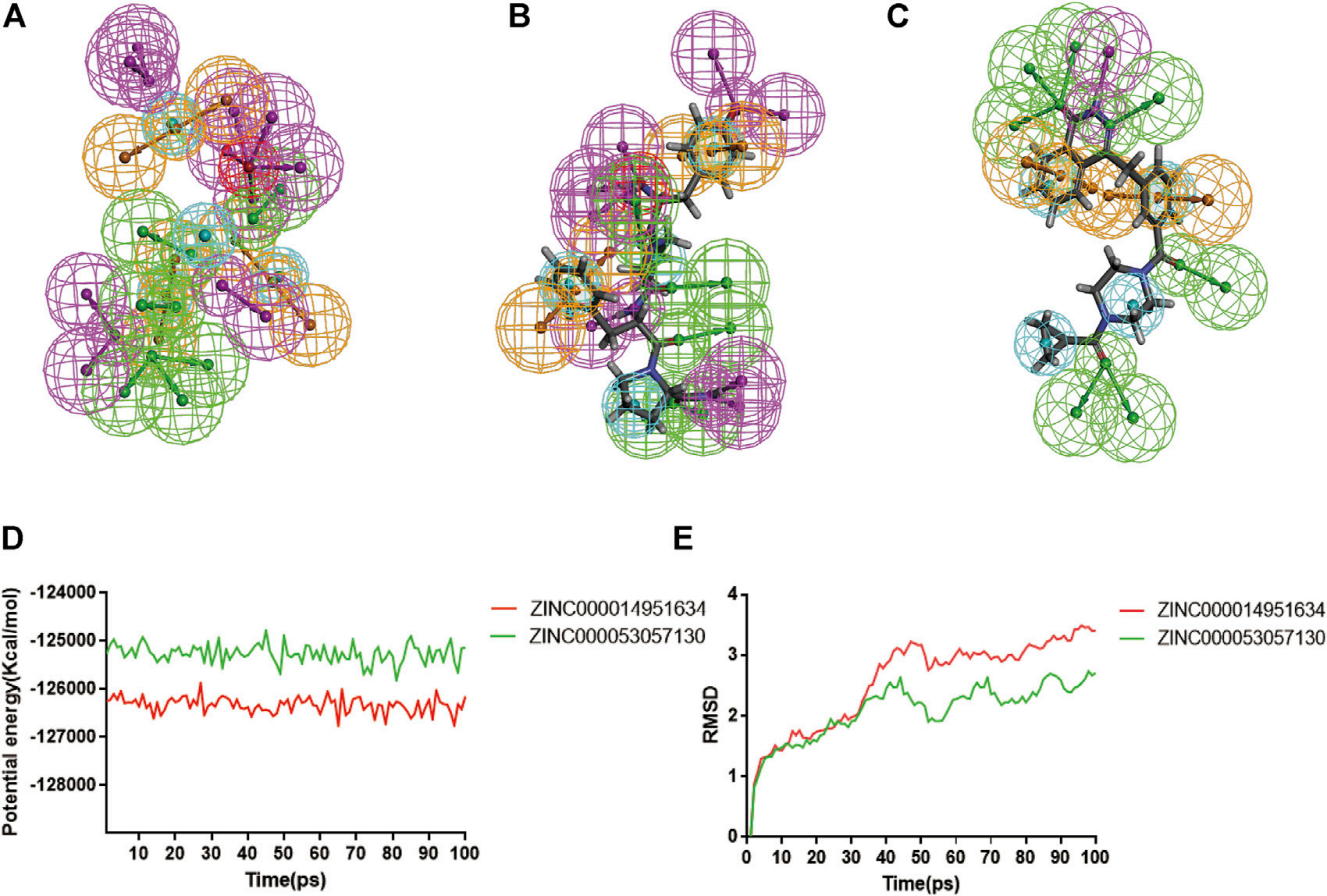
Pharmacophore predictions and molecular dynamics simulations of three complexes by DS 4.5. **(A)** ZINC000014951634: green represents hydrogen acceptor; blue represents the hydrophobic center; purple represents hydrogen donor; yellow represents aromatic ring; red represents inozable positive. **(B)** ZINC000053057130: green represents hydrogen acceptor; blue represents the hydrophobic center; purple represents hydrogen donor; yellow represents aromatic ring; red represents inozable positive. **(C)** Lynparza: green represents hydrogen acceptor; blue represents the hydrophobic center; purple represents hydrogen donor; yellow represents aromatic ring. **(D)** Potential Energy by molecular dynamics simulations of ZINC000014951634 and ZINC000053057130; **(E)** Average backbone RMSD of molecular dynamics simulations to ZINC000014951634 and ZINC000053057130.

According to the evaluation of feature pharmacophores by the 3D-QSAR module of DS 4.5, Compound 1 displayed eight hydrogen bond acceptors, ten hydrogen donors, four hydrophobic centres, and six aromatic rings, and one ionizable positive, respectively (Figure 8A). Compound 2 displayed seven hydrogen bond acceptors, nine hydrogen donors, four hydrophobic centres, four aromatic rings, and one ionizable positive, respectively (Figure 8B). In addition, Lynparza formed sixteen feature pharmacophores, including seven hydrogen bond acceptors, one hydrogen donor, four hydrophobic centres, and four rings aromatic respectively (Figure 8C).

Additionally, the molecular dynamics simulation module was carried out to assess the stabilities of the ligand-PARP-1 complexes in the natural environment. These complexes’ RMSD and potential energies were stable over time (Figures 8D, E). And the RMSD trajectory of each complex reached equilibrium after 70 ps So, hydrogen bonds and Pi-related interactions formed by compounds with PARP-1 may contribute to the stability of these complexes. And their complexes could exist in a natural environment stably as Lynparza.

## Discussion

Genomic instability is one of the most prevalent features of tumor cells, which offers therapeutic opportunities for glioma (Lord and Ashworth, 2012) (Hannigan et al., 2013). DNA damage repair (DDR) determines not only the occurrence and development of tumors but also the sensitivity or tolerance of tumor cells to radiotherapy, chemotherapy, and other treatments that induce DNA damage. PARP-1 was widely studied and played a key role in DNA repair pathways (Bryant et al., 2005; Brown et al., 2017). So, it is significant to explore PARP-1’s role in the prognosis prediction of glioma patients and develop more effective treatments.

In this study, we first systematically analyzed the influence of PARP-1 on DNA damage repair, prognosis, and chemoradiotherapy sensitization of glioma. 278 genes of the DNA damage repair proteins were mainly enriched in DNA repair, double-strand break repair, HDR through Homologous Recombination (HRR), Global Genome Nucleotide Excision Repair (GG-NER), ‘DNA repair pathways, full network’. Additionally, the expression levels of 278 DNA repair-related protein genes were significantly higher in PARP1_H than that in PARP1_L, which proved that PARP-1 matters a lot in the DNA repair pathway. Furthermore, all glioma patients, patients with radiotherapy or chemotherapy, and patients with both radiotherapy and chemotherapy had a better prognosis in PARP1_L than PARP1_H. According to the theory of combined lethality, radiotherapy and chemotherapy cause DNA damage in tumor cells, combined with the inhibitory effect of PARP-1 on DNA repair, resulting in a more potent cytotoxic effect on tumor cells (Shen et al., 2013). Therefore, PARP-1 is expected to be an evaluation indicator for the prognosis of patients. Inhibition of PARP-1 can improve the prognosis of glioma and promote chemoradiotherapy sensitization, which offers new ideas for treating glioma.

Next, we analyzed their DEGs between PARP1_H and PARP1_L. There were seven DEGs, including CCNA1, CLSPN, DTL, MGMT, POLN, SFN, and XRCC2. And the High group of each DEG had a worse OS than that of the Low group (Log-Rank test, CCNA1: *p* < 0.001, CLSPN: *p* < 0.001, DTL: *p* < 0.001, MGMT: *p* = 0.03, POLN: *p* < 0.001, SFN: *p* < 0.001, XRCC2: *p* < 0.001). The abnormal methylation of the promoter of the CCNA1 gene is closely related to the occurrence, growth, invasion, and metastasis of malignant tumors, such as cervical cancer (Yang et al., 2009). But it has not been reported in glioma. In addition, CLSPN regulates the cell G0/G1 phase cycle by the P53-p21/p27 molecular signaling pathway, thereby affecting the proliferation of glioblastoma. CLSPN may be a potential therapeutic target for glioblastoma. DTL is a protein predominantly expressed in the nucleus. It is a potential target for breast cancer, liver cancer, colorectal cancer, etc., but its role in glioma has not been reported (Baraniskin et al., 2012; Zhu et al., 2016; Li et al., 2022). MGMT promoter methylation is essential to evaluate glioma patients’ sensitization to alkylating agents for personalized and precise treatment and evaluate prognosis (Mansouri et al., 2019). POLN encodes a DNA polymerase type-A family member, which plays a role in DNA repair and homologous recombination. And it was first identified as a potential target for glioma. SFN (Stratifin), a cell cycle checkpoint protein, has been reported to be involved in tumorigeneses such as ovarian and nasopharyngeal cancer. Higher SFN expression was associated with significantly poorer overall survival (Hu et al., 2019; Zhang et al., 2019). XRCC2, a novel oncogene, is significantly overexpressed in glioma and can lead to poor prognosis in glioma patients (Liu et al., 2021). In summary, CCNA1, DTL, and SFN could serve as a new biomarker for glioma diagnosis, treatment, and prognosis evaluation.

Subsequently, a PARP1-related DPS was developed. CLSPN, MGMT, POLN, and SFN were identified as hub genes in our DPS by LASSO Cox regression. Furthermore, univariate and multivariate Cox analyses demonstrated that the four-gene DPS was an independent prognostic factor. Moreover, a predicting nomogram was constructed based on the PARP1-related DPS with an AUC value of 0.765 for predicting 1, 3, and 5-years patient survival.

Additionally, PARP-1 was proved an effective target for glioma therapy. So, a series of computer-aided techniques, including Discovery Studio 4.5, Schrodinger, and PyMol, were applied for virtual screening of favorable PARP-1 inhibitors. Lynparza was chosen as a reference inhibitor. Firstly, Libdock was performed between ligands and PARP-1 for virtual screening. Compounds with higher Libdock scores show better energy optimization and more stable conformations than others. The top 20 compounds by Libdock scores were chosen for subsequent pharmacological and toxicological analysis. Finally, ZINC000014951634 and ZINC000053057130 were shown to be no hepatotoxicity, non-CYP2D6 inhibitor, low Ames mutagenicity, low rodent carcinogenicity, and low developmental toxicity potential, which also strongly suggests their perspective application in drug development.

Additionally, to further evaluate ligand-protein complex affinity and stability, Molecular dynamics simulation and precise docking by CDOCKER were performed. Table 4 showed that the CDOCKER interaction energies of compounds 1 and 2 were significantly lower than that of the reference ligand Lynparza (−54.2416 kcal/mol), which indicated that these two compounds had higher stability and affinity with PARP-1 compared to Lynparza. Moreover, compound 1 and compound 2 formed several hydrogen bonds and Pi-related interactions as Lynparza. Furthermore, compounds 1, 2, and Lynparza interacted with PARP-1 by amino acid residues 861–988. The active position of the binding pocket provided a guide for PARP-1 targeted drug research and deep learning for PARP-1’s structure. In addition, compounds 1 and 2 were shown to have multiple pharmacophores, again suggesting the potential of compounds 1 and 2 as drugs. Moreover, according to the molecular dynamics simulation’s results, both potential energy and RMSD of these two complexes stabilized with time, validating the stabilities of the ligand-PARP-1 complexes in the natural environment.

Last but not least, this study provided new insight into the treatment and prognosis of glioma. Although this study was well designed and accurately measured, we acknowledge that this study still has some limitations. More prospective studies are needed to validate our results. And drugs need clinical trials to validate the specificity of PARP-1 inhibition.

## Conclusion

In this study, PARP-1 was proved to be an evaluation indicator for the prognosis of patients. And inhibition of PARP-1 can improve the prognosis of glioma and promote chemoradiotherapy sensitization, which offers new ideas for treating glioma. Furthermore, we developed a novel nomogram to quantitatively predict patient survival based on PARP-1-related DPS. And CCNA1, DTL, and SFN were discovered as novel biomarkers for glioma diagnosis, treatment, and prognosis evaluation. PARP-1 was proved an effective target for glioma therapy based on the results above. So, a series of computer-aided techniques were applied for screening favorable PARP-1 inhibitors. ZINC000014951634) and compound 2 (ZINC000053057130) were selected as favorable inhibitors of PARP-1. In conclusion, this study provided new insight into the treatment and prognosis of glioma.

## Data Availability

The datasets presented in this study can be found in online repositories. The names of the repository/repositories and accession number(s) can be found in the article/Supplementary Material.
